# Clinician and Patient-reported Outcomes Are Associated With Psychological Factors in Patients With Chronic Shoulder Pain

**DOI:** 10.1007/s11999-016-4894-0

**Published:** 2016-06-29

**Authors:** Adrian Wolfensberger, Philippe Vuistiner, Michel Konzelmann, Chantal Plomb-Holmes, Bertrand Léger, François Luthi

**Affiliations:** 1 Faculty of Biology and Medicine, University of Lausanne, Lausanne, Switzerland; 2 Institute for Research in Rehabilitation, Clinique Romande de Réadaptation SuvaCare, Sion, Switzerland; 3 Department for Musculoskeletal Rehabilitation, Clinique Romande de Réadaptation SuvaCare, Sion, Switzerland; 4 Department of Physical Medicine and Rehabilitation, Orthopaedic Hospital, Lausanne University Hospital, Avenue Pierre Decker, 1011 Lausanne, Switzerland

## Abstract

**Background:**

Validated clinician outcome scores are considered less associated with psychosocial factors than patient-reported outcome measurements (PROMs). This belief may lead to misconceptions if both instruments are related to similar factors.

**Questions:**

We asked: In patients with chronic shoulder pain, what biopsychosocial factors are associated (1) with PROMs, and (2) with clinician-rated outcome measurements?

**Methods:**

All new patients between the ages of 18 and 65 with chronic shoulder pain from a unilateral shoulder injury admitted to a Swiss rehabilitation teaching hospital between May 2012 and January 2015 were screened for potential contributing biopsychosocial factors. During the study period, 314 patients were screened, and after applying prespecified criteria, 158 patients were evaluated. The median symptom duration was 9 months (interquartile range, 5.5–15 months), and 72% of the patients (114 patients) had rotator cuff tears, most of which were work injuries (59%, 93 patients) and were followed for a mean of 31.6 days (SD, 7.5 days). Exclusion criteria were concomitant injuries in another location, major or minor upper limb neuropathy, and inability to understand the validated available versions of PROMs. The PROMs were the DASH, the Brief Pain Inventory, and the Patient Global Impression of Change, before and after treatment (physiotherapy, cognitive therapy and vocational training). The Constant-Murley score was used as a clinician-rated outcome measurement. Statistical models were used to estimate associations between biopsychosocial factors and outcomes.

**Results:**

Greater disability on the DASH was associated with psychological factors (Hospital Anxiety and Depression Scale, Pain Catastrophizing Scale combined coefficient, 0.64; 95% CI, 0.25–1.03; p = 0.002) and social factors (language, professional qualification combined coefficient, −6.15; 95% CI, −11.09 to −1.22; p = 0.015). Greater pain on the Brief Pain Inventory was associated with psychological factors (Hospital Anxiety and Depression Scale, Pain Catastrophizing Scale combined coefficient, 0.076; 95% CI, 0.021–0.13; p = 0.006). Poorer impression of change was associated with psychological factors (Hospital Anxiety and Depression Scale, Pain Catastrophizing Scale, Tampa Scale of Kinesiophobia coefficient, 0.93; 95% CI, 0.87–0.99; p = 0.026) and social factors (education, language, and professional qualification coefficient, 6.67; 95% CI, 2.77–16.10; p < 0.001). Worse clinician-rated outcome was associated only with psychological factors (Hospital Anxiety and Depression Scale (depression only), Pain Catastrophizing Scale, Tampa Scale of Kinesiophobia combined coefficient, −0.35; 95% CI, −0.58 to −0.12; p = 0.003).

**Conclusions:**

Depressive symptoms and catastrophizing appear to be key factors influencing PROMs and clinician-rated outcomes. This study suggests revisiting the Constant-Murley score.

**Level of Evidence:**

Level III, prognostic study.

## Introduction

There are various factors that can affect the results of treatment after shoulder injuries and there is no general consensus regarding which are the most decisive [11, 16]. Studies have shown that biological issues may not be the most important factor. For instance, patient-reported outcome measurements (PROMs) generally are improved regardless whether the integrity of the rotator cuff is restored [29]. There is also no general consensus regarding to what degree psychological distress or social factors may affect medical or surgical treatments [21, 31, 32, 44]. In 2014, Dunn et al. [9] showed that pain was not related to cuff tear severity, but could be correlated with comorbidities, lower education level, and ethnicity. Studies also have shown that kinesiophobia and catastrophic thinking were the most important factors related to disability for patients with an upper-extremity-specific disability [8], while psychological distress affects patient-reported scores of shoulder function [32, 35]. However, Roh et al. [35] showed that the Constant-Murley score, the most popular clinician-rated tool, may not be related to psychological distress.

To date, the majority of research on this topic has focused on specific aspects (for instance, psychological rather than social or biological factors) and predominantly used subjective outcomes. There is a lack of studies analyzing potential biopsychosocial factors and their effect on outcome scores using clinician-rated measurements and subjective outcome scores (patient-reported). Comparing instruments that measure physical impairments such as strength and mobility (recorded by clinicians) with others measuring only subjective perceptions of pain and disability appears to be important. For instance, surgeons or physiotherapists who feel underskilled to deal with patients’ subjective perceptions might be tempted to prioritize measurements rated by experienced clinicians rather than those related to patients’ perceptions. Nevertheless, this belief may lead to misconceptions and give incorrect information if both instruments are related to similar nonbiological factors. Recently, Roh et al. [36] found that poor pain-coping strategies were associated with decreased grip strength and decreased ROM at 3 months after hand fractures. To date, with the exception of a study by Roh et al. [35], there is no research, to our knowledge, addressing this question in patients with shoulder problems.

Therefore, we asked the following questions: (1) In patients with chronic shoulder pain, what biopsychosocial factors are associated with PROMs? (2) What biopsychosocial factors are associated with clinician-rated outcome measurements?

## Patients and Methods

### Study Setting and Participants

We conducted a single-center retrospective study with a total of 158 patients. This study was done at the Clinique Romande de Réadaptation in the French-speaking part of Switzerland. Patients, mostly blue collar workers with shoulder injuries, were included between May 2012 and January 2015. Patients are referred from all of the French-speaking counties of Switzerland, which includes urban and industrial city centers or more rural regions [28]. The patients were sent to the rehabilitation hospital by surgeons, general practitioners, or insurance medical advisors when they had persistent (≥ 3 months) pain and functional limitations incompatible with returning to work (median, 9 months; interquartile range, 5.5–15 months). The aim of the therapeutic program was to manage patients using an interdisciplinary approach (physiotherapy with individual and group therapies, psychological sessions with a cognitive approach, social advice and vocational training) to reduce pain and disabilities and improve chances of returning to work (usual or adapted). The therapeutic program lasted 4 to 5 weeks (at least 2 to 3 hours of daily therapy, excluding weekends). All patients, hospitalized after shoulder injuries, were eligible for this study if they had no severe traumatic brain injury at the time of the accident (Glasgow coma Scale ≤ 8), had no spinal cord injury, were capable of judgment, and were not under legal custody. Patients were injured after traffic, leisure, or work (59%) accidents.

The electronic patients’ files allowed all patients with shoulder injuries to be identified. Shoulder injuries were classified in the following categories: rotator cuff/bursa injuries and other shoulder injuries (clavicular fracture, proximal humerus or scapula fracture, glenohumeral dislocation, and acromioclavicular pathology). Inclusion criteria were unilateral shoulder injury and being between 18 and 65 years old. Exclusion criteria were bilateral injuries, concomitant injuries in another location (eg, spine), presence of an upper limb neuropathy (major and minor), or any missing data. Patients unable to read and understand the available validated versions of the PROMs also were excluded (see below). The protocol was approved by the local medical ethics committee (CCVEM 041/07). The study was performed in accordance with the ethical standards in the 1964 Declaration of Helsinki.

### Contributing Factors

Potential contributory factors were collected from routinely administered scales and medical records for all patients, and these were divided into biological, psychological, and social factors. Biological factors include (1) diagnosis category (rotator cuff/bursa injuries versus others); (2) Abbreviated Injury Scale (AIS) score (minor injury versus others) [1]; (3) Cumulative Illness Rating Scale [25], which assesses medical comorbidities (range, 0–56); and (4) the biological subscale (ie, chronicity, diagnostic dilemma, symptom severity/impairment, therapeutic challenge, and life threats [20]) of the INTERMED tool (range, 0–15) [18, 27, 39].

Psychological factors consisted of (1) the presence or absence of psychiatric diagnosis by a senior psychiatrist according to the ICD-10 classification (anxiety disorder, depressive disorder, mixed anxious and depressive disorder, psychotic disorder, adjustment disorder, and personality disorder); (2) Tampa Scale for Kinesiophobia (range, 17–68) [22]; (3) Pain Catastrophizing Scale (range, 0–52) [40]; (4) Anxiety and Depressive scores of the Hospital Anxiety and Depression Scale (range, 0–21 for both scores) [49]; and (5) psychological subscale (range, 0–15) of the INTERMED tool (ie, restrictions in coping, psychiatric dysfunction, resistance to treatment, psychiatric symptoms, and mental health threat).

Social factors included (1) educational level (≤ 9 years compulsory schooling versus > 9 years); (2) qualified work (yes versus no); (3) ability to speak the native language of the clinic (ie, French versus other); (4) and social subscale (ie, restrictions in integration, social dysfunction, residential instability, and social vulnerability) of the INTERMED tool (range, 0–15).

Questionnaires regarding medical comorbidities are compulsory in all Swiss rehabilitation hospitals; the INTERMED has been used since 2003, and the other questionnaires since 2012 in our hospital. The PROMs were validated, translated versions (French and several foreign languages).

Other potential confounding factors accounted for included age, sex, and pain severity at admission.

### Outcomes

Outcomes (before and after treatment) were categorized into two categories: patient-reported and clinician-rated. The PROMs studied included measures of disability (restriction in activities and participation), pain, and impression of change, as measured by the DASH, the Brief Pain Inventory questionnaire, and the Patient Global Impression of Change measure, respectively.

The DASH score [17] is a commonly used PROM to assess upper limb and shoulder function (range, 0–100). It consists of 30 questions describing the pain, feelings about the pain, and discomfort felt in daily living activities. The outcome measure was the difference in DASH score between entry and discharge. The Brief Pain Inventory questionnaire [4, 42] is divided into two main subscales: pain severity score, combining four different numeric rating scales (scale, 0–10) and pain interference score, combining seven different numeric rating scales (scale, 0–10). The outcome measure was the difference in pain severity score between entry and discharge. The Patient Global Impression of Change measure [21, 44] is recommended as a global indicator of meaningful changes for patients with chronic pain [10]. Patients were asked, on a seven-point scale if their pain-associated disabilities had changed compared with their feeling on admission (1 = worse than ever; 2 = much worsened; 3 = slightly worsened, 4 = unchanged, 5 = slightly improved; 6 = much improved; 7 = completely improved). The score was treated as a binary outcome (scores of 6 or 7 = improved versus scores of 1 through 5 = not improved). Validated tools for outcomes measurements are available in French and several others languages.

Clinician-rated outcome was measured using the Constant-Murley score [6], which is a clinician-based tool assessing global functioning of the shoulder. The test is divided into four categories: pain (15 points), activities of daily living (20 points), ROM (40 points), and strength (25 points). It has been validated and described as relevant in the analysis of shoulder function [5]. The outcome measure was the difference in Constant-Murley score between entry and discharge.

### Data Collection

To minimize the measurement bias, all records were collected electronically, without transcription from paper to the data files. All validated questionnaires were given in French and in foreign languages. Despite availability of other versions, we could not cover the entire panel of languages spoken in our clinic. Switzerland has a large number of immigrant workers who speak numerous languages. This partially explains why many eligible patients were not included (selection bias). The INTERMED, a validated semistructured interview [18, 39], was done by experienced nursing staff (more than 100 interviews by each nurse). The Constant-Murley score was done by highly experienced physiotherapists, familiar with the score through regular training and clear instructions.

### Accounting for all Patients

**Fig. 1:**
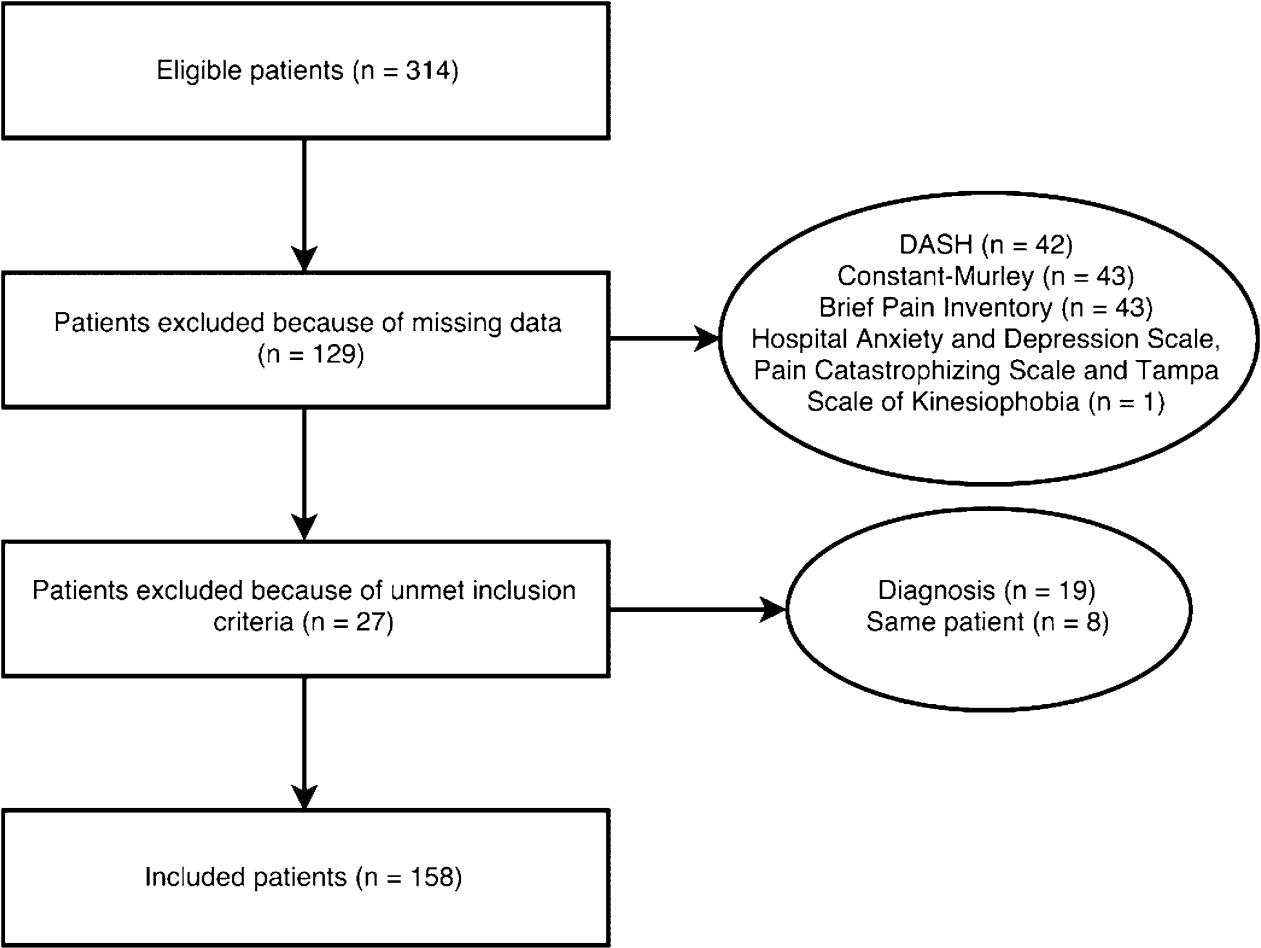
The flow chart shows the selection process used in the study. The final included number of patients corresponds to 50% of the eligible patients.

During the study period, 314 patients were eligible. Unmet inclusion criteria, missing data, and inability to read or understand few questionnaires led to disqualification of 156 subjects (Fig. 1).

The study participants were mostly middle-aged men (median, 48 years), with more than 1/2 being of foreign background, and 2/3 lacking a professional qualification. The majority were industry and construction workers (with mostly rotator cuff ruptures) (Table 1). No differences were observed between these two groups for most variables, except that the proportion of nonnative speakers, Hospital Anxiety and Depression Scale depression score, and INTERMED biological, physical, and social subscales were slightly higher in excluded patients (Table 2).

**Table 1:** Summary statistics

Type of variable	Variable	Possible values	Descriptive statistics
Outcomes	DASH at entry	0–100	51.8 (25.2)
	Constant-Murley score at entry	0–100	38.0 (23.8)
	Brief Pain Inventory at entry	0–10	4.5 (3.2)
	Patient Global Impression of Change	Not improved	101 (64%)
		Improved	57 (36%)
Biological	Age		48.0 (17.3)
	Cumulative Illness Rating Scale	0–56	4.0 (3.0)
	INTERMED biological	0–15	9.0 (1.3)
	Abbreviated Injury Scale	Minor	91 (58%)
		Moderate/serious	67 (42%)
	Diagnosis	Rotator cuff/bursa	114 (72%)
		Other	44 (28%)
	Sex	Male	129 (82%)
		Female	29 (18%)
Psychological	INTERMED psychological	0–15	3.0 (4.0)
	Hospital Anxiety and Depression Scale Anxiety	0–21	9.0 (7.0)
	Hospital Anxiety and Depression Scale Depression	0–21	6.0 (6.0)
	Pain Catastrophizing Scale	0–52	20.0 (18.5)
	Tampa Scale of Kinesiophobia	17–68	44.0 (11.0)
	Psychiatric diagnosis	Present	19 (12%)
		Absent	139 (88%)
Social	INTERMED social	0–15	5.0 (2.0)
	Education (years)	≤ 9	91 (58%)
		> 9	67 (42%)
	Native language	French	68 (43%)
		Other	90 (57%)
	Professional qualification	Present	52 (33%)
		Absent	106 (67%)

Possible values = the range for continuous variables and categories used for binary variables; descriptive statistics = median value and interquartile range for continuous variables and absolute number and relative number for binary variables; age and sex = adjusting variables.

**Table 2:** Measures comparison of included versus excluded participants

Measure	Included (n = 158)		Excluded (n = 146)		p value
DASH at entry	49.6 ± 17.3		54.8 ± 17.7		0.016
Constant-Murley score at entry	39.2 ± 15.6		37.2 ± 15.7		0.323
Brief Pain Inventory at entry	4.4 ± 2.2		4.8 ± 2.1		0.199
Patient Global Impression of Change (categories)	Improved	Not improved	Improved	Not improved	0.033
Number of patients	57 (36%)	101 (64%)	37 (25%)	112 (75%)	
Age (years)	47.1 ± 11.1		48.1 ± 10.1		0.406
Cumulative Illness Rating Scale	4.7 ± 2.5		5.0 ± 2.2		0.458
INTERMED biological	8.6 ± 1.4		9.4 ± 1.4		< 0.001
Abbreviated Injury Scale (categories)	Minor	Moderate or serious	Minor	Moderate or serious	0.402
Number of patients	91 (58%)	67 (42%)	69 (53%)	62 (47%)	
Sex (categories)	Men	Women	Men	Women	0.854
Number of patients	129 (82%)	29 (18%)	118 (81%)	28 (19%)	
INTERMED psychological	3.3 ± 2.6		4.5 ± 3.0		< 0.001
Hospital Anxiety and Depression Scale - Anxiety	8.9 ± 4.3		9.8 ± 4.6		0.109
Hospital Anxiety and Depression Scale -Depression	6.4 ± 3.9		7.7 ± 4.7		0.017
Pain Catastrophizing Scale	21.4 ± 12.5		23.5 ± 12.3		0.241
Tampa Scale of Kinesiophobia	44.9 ± 7.8		45.7 ± 8.2		0.369
INTERMED social	5.2 ± 1.7		5.8 ± 2.1		0.005
Education (years)	> 9	≤ 9	> 9	≤ 9	0.052
Number of patients	67 (42%)	91 (58%)	41 (31%)	90 (69%)	
French native language	Yes	No	Yes	No	0.020
Number of patients	68 (43%)	90 (57%)	39 (30%)	92 (70%)	
Professional qualification	Yes	No	Yes	No	0.392
Number of patients	52 (33%)	106 (67%)	37 (28%)	94 (72%)	

Quantitative values = mean ± SD.

### Statistical Method

Linear regression models were used to estimate the associations between the biological, psychological, and social factors and the continuous outcomes. Logistic regression was used when considering the Patient Global Impression of Change.

All independent variables were first screened individually, and then adjusted for confounding variables: age, sex, and pain severity at admission (except when the outcome was the change in pain).

Because some of the continuous variables were positively correlated, we built new variables as their geometric mean. Binary variables that were associated between each other also were grouped as new binary variables. This reduces the risk of collinearity in multivariable models.

All variables with a probability less than 0.10 were kept in the multivariable models. We then applied a backward elimination to select the final models as those which minimized the Akaike Information Criterion. All of these multivariable models contained the same adjusting variables as previously. A sample size consisting of 158 patients allows us to start the model selection procedure with up to 10 candidate factors (more than 15 observations per parameter) [14].

All analyses were performed with Stata^®^ 13.1 (StataCorp, College Station, TX, USA). The significance level was set as a probability less than 0.05.

## Results

Poor patient-reported outcomes are associated with psychological and social factors, but not biological factors, with the exception of the Brief Pain Inventory (Table 3). Greater disability on the DASH was associated with psychological factors (Hospital Anxiety and Depression Scale - anxiety, Hospital Anxiety and Depression Scale - depression, Pain Catastrophizing Scale combined coefficient, 0.64; 95% CI, 0.25–1.03; p = 0.002), and social factors (language, professional qualification combined coefficient, −6.15; 95% CI, −11.09 to −1.22; p = 0.015). Greater pain on the Brief Pain Inventory was associated with psychological factors (Hospital Anxiety and Depression Scale - anxiety, Hospital Anxiety and Depression Scale - depression, Pain Catastrophizing Scale combined coefficient, 0.076; 95% CI, 0.021–0.13; p = 0.006; and INTERMED biological score coefficient, 0.27; 95% CI, 0.05 to 0.49; p = 0.0180). Poorer impression of change (Patient Global Impression of Change) was associated with psychological factors (Hospital Anxiety and Depression Scale – anxiety and Hospital Anxiety and Depression Scale - depression anxiety, Pain Catastrophizing Scale, Tampa Scale of Kinesiophobia coefficient, 0.93; 95% CI, 0.87–0.99; p = 0.026) and social factors (education, language, and professional qualification coefficient, 6.67; 95% CI, 2.77–16.10; p < 0.001). Interestingly, no association between psychiatric diagnoses and poor patient-reported outcomes was observed.

**Table 3:** Patient-reported-outcomes analysis models

Outcome	Variable	Univariable coefficient (95% CI)	p value		Multivariable coefficient (95% CI)	p value
DASH	Cumulative Illness Rating Scale	0.39 (−0.51 to 1.29)	0.394			
	INTERMED biological	1.53 (−0.09 to 3.15)	0.063			
	Abbreviated Injury Scale	−0.47 (−4.53 to 4.44)	0.985			
	Diagnosis	2.28 (−2.70 to 7.27)	0.367			
	INTERMED psychological	0.23 (−0.62 to 1.08)	0.593			
	Hospital Anxiety and Depression Scale - Anxiety	0.63 (0.13–1.14)	0.015*	}	0.64 (0.25–1.03)	0.002*
	Hospital Anxiety and Depression Scale - Depression	1.03 (0.48–1.58)	**<** 0.001*			
	Pain Catastrophing Scale	0.30 (0.12–0.47)	0.001*			
	Tampa Scale of Kinesiophobia	0.15 (−0.14 to 0.44)	0.310			
	Psychiatric diagnosis	−2.62 (−9.40 to 4.16)	0.447			
	INTERMED social	0.78 (−0.47 to 2.03)	0.219			
	Years of education	−4.40 (−8.96 to 0.17)	0.059	}	−6.15 (−11.09 to −1.22)	0.015*
	Native language	−7.62 (−12.04 to −3.21)	0.001*			
	Professional qualification	−5.23 (−9.88 to −0.58)	0.028*			
Brief Pain Inventory	Cumulative Illness Rating Scale	0.05 (−0.08 to 0.18)	0.445			
	INTERMED biological	0.33 (0.10–0.55)	0.004*		0.27 (0.05–0.49)	0.018*
	Abbreviated Injury Scale	0.18 (−0.44 to 0.80)	0.559			
	Diagnosis	−0.05 (−0.74 to 0.64)	0.885			
	INTERMED psychological	0.08 (−0.04 to 0.20)	0.198			
	Hospital Anxiety and Depression Scale - Anxiety	0.10 (0.03–0.17)	0.005*	}	0.076 (0.021–0.13)	0.006*
	Hospital Anxiety and Depression Scale - Depression	0.12 (0.05–0.20)	0.002*			
	Pain Catastrophing Scale	0.03 (0.01–0.06)	0.011*			
	Tampa Scale of Kinesiophobia	0.01 (−0.03 to 0.05)	0.521			
	Psychiatric diagnosis	−0.20 (−1.15 to 0.74)	0.666			
	INTERMED social	0.16 (−0.01 to 0.34)	0.071			
	Years of education	−0.20 (−0.83 to 0.42)	0.522			
	Native language	−0.60 (−1.20 to 0.01)	0.055			
	Professional qualification	−0.46 (−1.11 to 0.19)	0.163			
Patient Global Impression of Change	Cumulative Illness Rating Scale	0.99 (0.86–1.14)	0.892			
	INTERMED biological	0.79 (0.61–1.01)	0.064			
	Abbreviated Injury Scale	0.58 (0.29 –1.19)	0.138			
	Diagnosis	0.74 (0.35–1.58)	0.434			
	INTERMED psychological	0.84 (0.73–0.96)	0.012*		0.86 (0.74–1.01)	0.069
	Hospital Anxiety and Depression Scale - Anxiety	0.92 (0.85–1.00)	0.044*	}	0.93 (0.87–0.99)	0.026*
	Hospital Anxiety and Depression Scale - Depression	0.87 (0.79–0.96)	0.006*			
	Pain Catastrophing Scale	0.94 (0.91–0.97)	< 0.001*			
	Tampa Scale of Kinesiophobia	0.94 (0.89–0.98)	0.007*			
	Psychiatric diagnosis	0.96 (0.32–2.82)	0.934			
	INTERMED social	0.86 (0.72–1.07)	0.184			
	Years of education	5.41 (2.55–11.47)	< 0.001*	}	6.67 (2.77–16.10)	< 0.001*
	Native language	8.90 (4.05–19.56)	< 0.001*			
	Professional qualification	4.05 (1.95–8.41)	< 0.001*			

Analysis done in linear regression for DASH and Brief Pain Inventory, in logistic regression for Patient Global Impression of Change; each association is adjusted for age and sex, and for the DASH for Brief Pain Inventory score at entry in the clinic; * = significant;

Similarly, a worse Constant-Murley score was associated with psychological factors (Hospital Anxiety and Depression Scale - depression, Pain Catastrophizing Scale, Tampa Scale of Kinesiophobia combined coefficient, −0.35; 95% CI, −0.58 to -0.12; p = 0.003), but not biological factors (Table 4). Social factors (education years, native language, and professional qualification coefficient, 3.37; 95% CI, −0.67 to 7.41; p = 0.101) met criteria for inclusion in our multivariate analysis, but was not found to be a contributory factor on final analysis.

**Table 4:** Clinician-rated-outcome analysis model

Outcome	Variable	Univariable coefficient (95% CI)	p value		Multivariable coefficient (95% CI)	p value
Constant-Murley score	Cumulative Illness Rating Scale	0.37 (−0.35 to 1.09)	0.308			
	INTERMED biological	−0.32 (−1.63 to 0.99)	0.627			
	Abbreviated Injury Scale	−1.69 (−5.28 to 1.90)	0.355			
	Diagnosis	−1.78 (−5.78 to 2.21)	0.380			
	INTERMED psychological	0.10 (−0.58 to 0.78)	0.768			
	Hospital Anxiety and Depression Scale - Anxiety	−0.26 (−0.67 to 0.15)	0.211			
	Hospital Anxiety and Depression Scale - Depression	−0.70 (−1.15 to −0.26)	0.002*	}	−0.35 (−0.58 to −0.12)	0.003*
	Pain Catastrophing Scale	−0.19 (−0.33 to −0.04)	0.012*			
	Tampa Scale of Kinesiophobia	−0.29 (−0.51 to −0.06)	0.013*			
	Psychiatric diagnosis	−0.19 (−5.62 to 5.23)	0.944			
	INTERMED social	0.03 (−0.98 to 1.03)	0.957			
	Years of education	3.69 (0.03–7.34)	0.048*	}	3.37 (−0.67 to 7.41)	0.101
	Native language	5.95 (2.40–9.50)	0.001*			
	Professional qualification	3.31 (−0.45 to 7.08)	0.084			

Analysis done in linear regression for Constant-Murley score; each association is adjusted for age, sex, Brief Pain Inventory score at entry in the clinic; * = significant (p value < 0.05).

## Discussion

The factors related to clinical changes and disability in patients with shoulder pain are variable and go well beyond biological and objective data [12, 21, 23, 32, 35]. Psychological and social factors may play an important mediating role between physical impairments and perceived disability [24]. Consequently, the choice of tools measuring these parameters may have important implications in interpreting results [47]. Therefore, we aimed to assess whether, in patients with chronic shoulder complaints, psychological and social variables were associated with clinical improvement and disability as opposed to biological factors. The results of this study suggest that psychological factors are associated with clinician-rated and patient-reported outcomes, whereas biological factors are not. Social variables are associated only with the DASH and Patient Global Impression of Change score.

Our study had numerous limitations. First, the unusual setting may induce a limitation in generalization of the results (for details, see Methods). However, our focus was on middle-aged patients with chronic shoulder pain (rotator cuff tears, 72%), who may have difficulty returning to heavy work. The type of rehabilitation hospital, such as our clinic, is relatively common throughout Europe. The selection bias with an exclusion rate of 49% of eligible patients is partly attributable to the retrospective design of the study. Another reason that explains the bias was the lack of validated questionnaires for several nonnative speakers. In additional, our electronic data medical records previously were not calibrated to signal missing or aberrant values and we decided to include only patients with complete data. However, only minor differences in the investigated variables were found between included and excluded patients (Table 2). Moreover, notwithstanding the great number of available factors, it is likely that other potential variables of interest were not included in this study, such as self-efficacy, health literacy, and social support [2, 19, 37]. Possible important biologic factors (integrity of rotator cuff repair, glenohumeral arthritis) also were not reported. In addition, no consideration was given to the role of workers’ compensation in our study; compensation has been reported to have a profound role in outcomes [15]. The lack of consideration is attributable to the Swiss insurance framework. Health and accident insurances are compulsory in Switzerland and patients are insured against occupational and nonoccupational injuries. The insurer must pay for medical treatment as long as substantial improvement can be anticipated. The insured persons have legal rights for integration measures, but they are obliged to cooperate and do everything possible to return to occupational activity, avoiding the need for pension [46]. Therefore it was not possible to use workers’ compensation as a contributing factor. Even though we studied widely used shoulder outcome measurements, some others instruments like the Simple Shoulder Test [26], or the American Shoulder and Elbow Surgeons score [33] were not evaluated. Nevertheless, results of former studies using these outcome measurements were in line with our results [32, 35]. Finally, owing to the study design, only associations were found and no conclusions could be drawn regarding a possible causative effect.

Higher level of depression symptoms, catastrophizing thoughts, and to a lesser extent, fear of movement, fear of pain, and anxiety were related to higher disability, greater pain severity, and lowest perceptions of clinical improvement; and similarly, social factors also were contributory. Depression and pain catastrophizing, generally considered poor prognostic factors, have been investigated in patients with lower-back pain [30, 48], but much less in patients with upper extremity disability [12, 34]. Depressive symptoms have been well-described as independent factors related to the DASH, American Shoulder and Elbow Surgeons score, and Simple Shoulder Test [3, 35]. In other studies [31, 32], distress rather than depression has been investigated using instruments combining depressive and somatic symptoms. Higher level of psychological distress was associated with higher pain and lower function before shoulder arthroscopy. Psychiatric comorbidities were not related to PROMs (Table 3). The binary character of psychiatric diagnoses may not accurately describe the psychological status of patients and may explain the lack of sensitivity. However, to our knowledge, there are no published studies exploring the relationship between psychiatric comorbidities and shoulder outcome measures. Concerning the suggested role of social factors, a lower educational level was found as an independent factor associated with pain severity after atraumatic rotator cuff tears [9], supporting the strong association shown in the medical literature between lower levels of education and poor health outcomes [43]. Nonnative patients may have different cultural representations and expectations, which also might affect health outcomes [38].

Finally, the almost complete absence of association between outcomes and biological factors may be attributable to the lack of some potential others biological contributing factors (limitations above).

Higher level of depression symptoms, catastrophizing thoughts, and fear of movement were related to poorer outcome measured with the Constant-Murley score. These psychological factors are all components of the Fear-Avoidance Model, one of the most popular theoretical frameworks in chronic pain [45]. So far, the relationship between the Constant-Murley score and depression (measured with the Center for Epidemiologic Studies-Depression scale) was measured in only one study, with no association being shown [35]. However, it cannot be ruled out that use of different depression scales might achieve different associations, and in the aforementioned study, patients were less disabled than our patients were, making the relationship potentially dependent on level of disability. Physical measurements may be disturbing for patients with chronic pain, and shoulder apprehension testing was shown to induce specific reorganization in brain areas involved in motor resistance, motor behavior, anxiety, and emotional regulation [7, 13]. Our results, and those of Roh et al. [36], challenge beliefs that outcomes rated by experienced professionals may be less influenced by psychological factors.

The results of our study suggest that psychological factors are associated with clinician and patient shoulder outcome instruments, and consequently, should be screened appropriately (particularly depression and catastrophizing). How the patient presents therefore can influence the clinician’s judgement [41]. Careful consideration should be given to this element when evaluating outcomes measurements. Additional research in various settings may be needed to improve knowledge of the complex interactions between patients and clinicians during outcome evaluation [24]. It also would be advisable to revisit the Constant-Murley score, studying which components are influenced by psychological factors.
